# Antifungal Properties of Crude Extracts, Fractions, and Purified Compounds from Bark of *Curatella americana* L. (Dilleniaceae) against *Candida* Species

**DOI:** 10.1155/2015/673962

**Published:** 2015-08-11

**Authors:** Cleyton Eduardo Mendes de Toledo, Patrícia Regina Santos, João Carlos Palazzo de Mello, Benedito Prado Dias Filho, Celso Vataru Nakamura, Tânia Ueda-Nakamura

**Affiliations:** ^1^Laboratory of Microbiology Applied to Natural and Synthetic Products, State University of Maringá, Colombo Avenue 5790, 87020-900 Maringá, PR, Brazil; ^2^Faculty of Medicine and Pharmacy, Ingá College, Rodovia PR317, 6114, 87035-510 Maringá, PR, Brazil; ^3^Laboratory of Pharmacognosy-Palafito, State University of Maringá, Colombo Avenue 5790, 87020-900 Maringá, PR, Brazil

## Abstract

The ethnomedicinal plant *Curatella americana* L. (Dilleniaceae) is a common shrub in the Brazilian cerrado, in which crude extract showed antifungal activity in a preliminary study. In this work, the antifungal and cytotoxic properties of the crude extract, fractions, and isolated compounds from *C. americana* were evaluated against the standard yeast strains *Candida albicans*, *C. tropicalis*, and *C. parapsilosis*, clinical isolates, and fluconazole-resistant strains. The combinatory effects between subfractions and isolated compounds and effects on cell morphology, virulence factors, and exogenous ergosterol were also evaluated. The MIC obtained against the *Candida* species including fluconazole-resistant strain ranged from 15.3 to 31.3 *µ*g/mL for crude extract, 3.9 to 15.6 *µ*g/mL for ethyl acetate fraction, and 7.8 to 31.3 *µ*g/mL for subfractions. The isolated compounds identified as 4′-*O*-methyl-catechin, epicatechin-3-*O*-gallate, and 4′-*O*-methyl-catechin-3-*O*-gallate showed lower antifungal activity than the crude extract and fractions (MIC ranging from 31.3 to 125.0 *µ*g/mL). The addition of exogenous ergosterol to yeast culture did not interfere in the antifungal activity of the extract and its fractions. Synergistic antifungal activity was observed between subfractions and isolated compounds. The effects on virulence factors and the different mechanisms of action compared to fluconazole and nystatin suggest that this ethnomedicinal plant may be an effective alternative treatment for candidiasis.

## 1. Introduction

In the Brazilian cerrado there is a large and still unknown biodiversity [1]. Studies of various plant species with therapeutic use have been carried out, and many compounds with biological activity were obtained from medicinal plants of the Brazilian cerrado [2–4]. Considering the antimicrobial activity, several studies have been conducted [5] to investigate the potential use of herbal drugs in the treatment or control of infectious diseases searching for drugs with fewer side effects, lower costs, and broad-spectrum. This approach is also relevant in case of diseases caused by opportunistic and drug-resistant microorganisms, mainly in immunocompromised patients [6].

The species* Curatella americana* L. (Dilleniaceae) is a very common shrub in the Brazilian cerrado, and it is also characteristic of the Neotropical savanna, occurring from southern Mexico to Bolivia [7]. The plant stem bark, popularly known as “Lixeira” and “Sambaíba,” is used in folk medicine due to its anti-inflammatory and antiulcer effects in the treatment of wounds and other skin diseases [8–10].

Fungal infections of the oral cavity are caused by a group of saprophytic fungi that includes eight species of the genus* Candida*.* Candida albicans* is the most common species that resides in the oral cavity in humans, accounting for 70–80% of oral isolates.* Candida parapsilosis*,* C. tropicalis*, and* C. glabrata* comprise approximately 10% of oral isolates [11].* Candida albicans* is also the main cause of vaginal candidiasis, a common mucosal infection that occurs with the highest prevalence in women aged 20–30 years [12]. Because of the resistance of yeast pathogens to pharmacotherapeutics, candidiasis therapy frequently fails. The resistance of* Candida* species to azole antifungals is the most prevalent type of resistance to antifungals [13]. Researchers have shown that 3.6% of* C. albicans* vaginal isolates were found to be resistant to fluconazole, and 16.2% were resistant to itraconazole [14, 15]. In addition to resistance to antifungals, fungal infections caused by opportunistic pathogens have become more frequent, partially as a result of prolonged antibiotic therapy and the increasing number of immunocompromised patients [16]. Therefore, developing therapeutic strategies for the treatment of candidiasis in immunocompromised patients is particularly important, with the goal of developing drugs with better effectiveness, reduced side effects, and lower cost. The price of antifungal agents is an important factor when considering that the prevalence of opportunistic infections also depends on economic factors, involving health, hygiene, and nutritional status. We performed a survey of plants that are commonly used in traditional medicine, and extracts from the stem bark of* C. americana* had very promising antifungal activity [17]. Thus, considering obtaining a possible alternative for the treatment of oral and vaginal candidiasis, the present study evaluated the antifungal properties of extracts, fractions, and isolated compounds from the bark of the plant species* Curatella americana* (L.).

## 2. Materials and Methods

### 2.1. Plant Material

The stem bark from* Curatella americana* L. was collected at the Tocantins cerrado (altitude 267 m; S 10°11.884′; HO 48°17.714′) under authorization number 14043-1 from the Instituto Brasileiro do Meio Ambiente e dos Recursos Naturais Renováveis (IBAMA). Identification occurred at the Tocantins Herbarium, Universidade Federal de Tocantins, by Dr. Solange F. Lolis. Voucher specimens were deposited in the same herbarium (registration HTO2234).

### 2.2. Preparation of Crude Extract and Fractionation

The stem bark was dried under shade in the open air to reduce deterioration of the plant drug material. It was then comminuted and dried at room temperature in the dark. After drying, the plant material was powdered (Tigre ASN5), and the crude extract (CE; 10% w/v) was obtained by turboextraction according to Toledo et al. [17] using acetone/water (7 : 3, v/v) as the extractor liquid. The lyophilized CE was then dissolved in acetone/water (2 : 8, v/v) and partitioned with ethyl acetate to obtain a water fraction (F1) and an ethyl acetate fraction (F2). The F2 fraction (2 g) was chromatographed on a Sephadex LH-20 column (*h* = 750 mm, *Ø* = 55 mm) using 50% ethanol (4 L), absolute ethanol (1 L), and acetone/water (7 : 3, v/v; 2 L) as the eluent system. Nineteen fractions were obtained, in which the most active (F2#5 and F2#6) were subfractionated by high-speed countercurrent chromatography (HSCCC). For identification, the isolated compounds were acetylated, and their nuclear magnetic resonance spectra were determined on a Varian instrument (Mercury 300BB, 300 MHz for ^1^H, 75 MHz for ^13^C) using TMS as an internal standard. The spectra were analyzed and compared with data from the literature [18–21].

### 2.3. Evaluation of Antifungal Properties

The standard yeast strains* Candida albicans* (ATCC 10231) and* C. parapsilosis* (ATCC 22019; supplied by Fundação Oswaldo Cruz, Rio de Janeiro, Brazil) were maintained at 4°C on Sabouraud dextrose agar plates and subcultured at 37°C in Sabouraud dextrose broth before each experiment to ensure viability and purity. The yeast strains evaluated in the present study also included three isolates from vaginal fluid (*C. albicans*,* C. parapsilosis*, and* C. tropicalis*) obtained from women with no previous history of immunodeficiency, four strains that were resistant to fluconazole (*C. albicans* 32res, 32Bres, 48res, and 103res) and one susceptible strain (*C. albicans* 48sen). All of these isolates are part of the yeast culture collection at our laboratory [22]. The MICs of the crude extracts were determined against the yeast using broth microdilution techniques (CLSI, 2008). Nystatin (Sigma) and fluconazole (kindly donated by Pfizer) were used as a control. The assays were done in triplicate in three independent experiments.

### 2.4. Cytotoxicity Assay

A suspension of Vero cells (ATCC CCL-81) was counted in a Neubauer hemocytometer and diluted in Dulbecco's Modified Eagle Medium (DMEM) that contained 10% fetal bovine serum (FBS) and 50 *μ*g/mL gentamycin to obtain a suspension of 2.0 × 10^5^ cells/mL. One hundred microliters of cell suspension were placed in each well of 96-well plate and incubated at 37°C with 5% CO_2_ for 24 h or until formation of confluent cell monolayers. The pure compound was initially dissolved in dimethyl sulfoxide (DMSO; final concentration, 1%) and then in DMEM to obtain the initial solution that was subsequently diluted in DMEM and added to each cell monolayer. The negative control consisted of the addition of only the medium and antibiotics. The 96-well plates were incubated for 72 h in a humidified chamber at 37°C with 5% CO_2_. Cell viability was determined by the sulforhodamine B colorimetric method [23]. The results were obtained using an enzyme-linked immunosorbent assay (ELISA) reader (Bio-Tek Power Wave XS Microplate Fluorescence Reader) at an optical density (OD) of 530 nm. Cytotoxicity is expressed as a percentage of the OD of the control. The assays were done in triplicate in three independent experiments.

### 2.5. Hemolysis Assay

Human erythrocytes from a healthy donor (type A^+^) were collected and washed with saline solution that contained 1% glucose, buffered to pH 7.25 (SSG). To determine hemolytic activity, a 3% erythrocyte suspension was incubated for 2 h with the crude extracts in 96-well microplates at 37°C (tested concentrations: 15.6, 31.3, 62.5, 125.0, 250.0, 500.0, and 1000 *μ*g/mL). Hemolysis was determined by reading the absorbance of the supernatant from each well using a spectrophotometer at 550 nm. The positive control (100% hemolysis) and negative control (0% hemolysis) were also evaluated by incubating the erythrocytes with 1% Triton X-100 in SSG and SSG alone, respectively [24, 25]. The assays were done in triplicate in three independent experiments.

### 2.6. Evaluation of Synergistic Effect: Checkerboard Test

Checkerboard tests were performed using the CLSI [26] broth microdilution reference procedure with a final inoculum of 0.5–2.5 × 10^3^ CFU/mL and RPMI 1640 medium buffered with 0.165 M MOPS. The final concentrations were 1.6–100 *μ*g/mL for fluconazole, 0.8–50.0 *μ*g/mL for nystatin, and 0.5–500 *μ*g/mL for the CE, fractions, and subfractions. The plates were incubated at 37°C for 48 h, and the endpoints were defined as the lowest concentration of antifungal agent that resulted in the total inhibition of visual growth. The fractional inhibitory concentration (FIC) index is defined as the sum of the MIC of each drug in combination, divided by the MIC of the drug used alone. An FIC index <0.5 is considered synergism. An FIC index >4 is considered antagonism. An FIC index >0.5 but <4 is considered indifferent [27]. The assays were done in triplicate in three independent experiments.

### 2.7. Effect of Exogenous Ergosterol on the MIC

To determine whether the effects of the CE, fractions, and subfractions on yeast are attributable to their interaction with ergosterol, a competition assay using ergosterol during the MIC determination assay was performed. The MIC was determined according to the CLSI [26] guidelines described above in the presence and absence of several concentrations (50, 100, and 200 *μ*g/mL each) of ergosterol (Sigma). Amphotericin B was used as a control drug. The MIC was read at 24 h according to the growth of the control fungus. The assays were done in triplicate in three independent experiments.

### 2.8. Effect on Virulence Factors

*Effect on C. albicans Budding.* Different concentrations of CE and subfractions diluted in RPMI 1640 medium were tested to assess the effect on* C. albicans* budding. Cells (0.5–2.5 × 10^3^ CFU/mL) were incubated at 37°C for 48 h and observed using a negative-staining technique with 7% aqueous nigrosin. Three hundred cells were counted in each smear by light microscopy, and the percentage of budding cells was calculated [28].

*Effect on Germ-Tube Formation.* A yeast suspension of 1.0 × 10^6^ CFU/mL was treated (MIC, sub-MIC, and 2x MIC) with the CE and subfraction diluted in sterile fetal bovine serum (FBS) and observed by phase-contrast microscopy (Zeiss Axiovert 25) after 2-3 h incubation at 37°C. Nystatin was used as the standard antifungal drug. For quantification, the cells were considered germinated if they had a germ tube at least twice the length of the cell [29].

*Evaluation of Adherence Inhibition on a Glass Surface.* The yeasts were treated with different concentrations (MIC, sub-MIC, and 2x MIC) of CE, subfractions, and nystatin for 24 h at 37°C, washed with 10 mM phosphate-buffered saline (PBS) at pH 7.2, and counted in a Neubauer chamber to obtain 1.0 × 10^6^ CFU/mL. A 500 *μ*L aliquot of the yeast suspension was added on cover slips alone. The interaction between the yeast and the abiotic surface was performed for 1 h at 37°C. The cover slips were then washed in 10 mM PBS, pH 7.2. In the analysis of yeast adherence to the abiotic surface, 20–100 fields were counted with a 100x objective [22].

*Evaluation of Effect of the Extracts on the Ability to Adhere to Buccal Epithelial Cells (BECs).* Samples of* C. albicans* were grown in medium with glucose for 24 h at 37°C. They were then washed twice with PBS (pH 7.2) and resuspended in PBS, adjusting the concentration to 1.0 × 10^7^ cells/mL. We collected BECs from a healthy volunteer using sterile swab, which were shaken in PBS and washed twice with PBS to remove possible microorganisms. The cells were resuspended in PBS, adjusting the concentration to 1.0 × 10^5^ cells/mL. The yeasts were treated with the drugs (MIC, sub-MIC, and 2x MIC) for 1 h at 37°C under agitation. A 500 *μ*L volume of the epithelial cell suspension (1.0 × 10^5^ cells/mL) was added to 500 *μ*L of treated and untreated yeast (1.0 × 10^7^ yeast/mL) and incubated for 30 min at 37°C. The suspension was then filtered through a polycarbonate membrane (12 *μ*m, 47 mmØ, Millipore, Billerica, MA, USA), which was washed with PBS to remove the nonadhered yeast. The cells were then fixed with methanol, stained with crystal violet solution, and observed under an optical microscope. The number of treated yeast that adhered to 100 epithelial cells was counted and compared with untreated yeast [30].

*Effect on Cell Surface Hydrophobicity (CSH).* Yeasts that were treated with the CE and subfraction (MIC and sub-MIC) were washed with 50 mM PBS (pH 7.4) and resuspended in the same buffer. Turbidity was determined in a spectrophotometer at 660 nm. The cell suspensions were then mixed with xylene 2.5 : 1 (v/v; Merck) and shaken for 2 min. The tube was left for 20 min at room temperature to obtain phase separation. The turbidity of the aqueous phase was read at 660 nm. The hydrophobicity index (HI) was calculated as HI = (*A*_control_ − *A*_test_) × 100/*A*_control_, where *A*_control_ indicates the OD of the strains before xylene treatment and *A*_test_ indicates the OD of the strains after xylene treatment [31]. The assays were done in triplicate in three independent experiments.

### 2.9. Scanning Electron Microscopy

*C. albicans* treated with the CE and subfractions were fixed with 2.5% glutaraldehyde for 2 h at room temperature. After fixation, small drops of the sample were placed on a specimen support with poly-L-lysine. Subsequently, the samples were dehydrated in graded ethanol, critical-point-dried in CO_2_, coated with gold, and observed in a Shimadzu SS-550 scanning electron microscope.

### 2.10. Evaluation of Oxygen Consumption

To evaluate oxygen uptake [32], stationary-phase cells (approximately 1.5 × 10^8^ cells/mL) were grown with and without the test drugs in RPMI 1640 broth at 37°C overnight, harvested, washed with 0.025 M PBS (pH 7.2), and resuspended in 0.025 M phosphate buffer (pH 7.2) at a cell density of 5.0 × 10^8^/mL. Oxygen uptake measurements were made at 30°C using a Clark-type oxygen electrode. Oxygen uptake rates were calculated as *n*moles of oxygen consumed/60 s/10^8^ cells, and the results are expressed as a percentage relative to controls (untreated yeast). The assays were done in triplicate in three independent experiments.

## 3. Results and Discussion

The phytochemical analysis of the bark of* C. americana* L. has demonstrated the presence of compounds as flavonoids, terpenes, phenolics, saponins, and steroids that have also been isolated in other species of the Dilleniaceae family [33]. Among the phenolic compounds, tannins have been found in considerable amounts. Researchers identified oligomeric and polymeric proanthocyanidins by high-performance liquid chromatography and discussed the possibility that they are responsible for its gastroprotective effect [34]. In the present work, from 1 kg of the dried and powdered bark of* C. americana* and 10 L of acetone/water (7 : 3, v/v), we obtained 148.79 g of dried extract (CE; 14.88% yield). From the liquid-liquid partition from the CE (120 g), we obtained 72.03 g of the aqueous phase (60.03% yield) and 34.17 g of the ethyl acetate phase (F2; 28.48% yield). From the ethyl acetate fraction F2 (20.00 g), we obtained 19 subfractions by column chromatography (Sephadex LH-20), of which the highest yields and best antifungal activity were obtained for F2#5 (3.21 g), F2#6 (2.47 g), and F2#7 (1.68 g). Using HSCCC, we obtained 4′-*O*-methyl-catechin (F2#5.4.1) and F2#5.2.2 (unidentified substance) from F2#5 and epicatechin-3-*O*-gallate (F2#6.2.1) and 4′-*O*-methyl-catechin-3-*O*-gallate (F2#6.1.2) from F2#6.

The antifungal activity of the crude extract, fractions, and subfractions obtained by broth microdilution techniques is shown in Table 1. The F2 fractionation by column chromatography yielded 19 subfractions monitored by TLC (Thin Layer Chromatography). Of these, the subfractions F2#5 and F2#6 had the lowest MIC against* C. albicans* (31.3 and 15.6 *μ*g/mL, resp.). The isolated compounds had greater MICs than the subfractions, fractions, and extracts, ranging from 31.3 to 125.0 *μ*g/mL. In the tests that evaluated fungicidal activity, we observed the growth of yeast cells after drug removal, demonstrating that the CE and its fractions had fungistatic activity.

**Figure 1:**
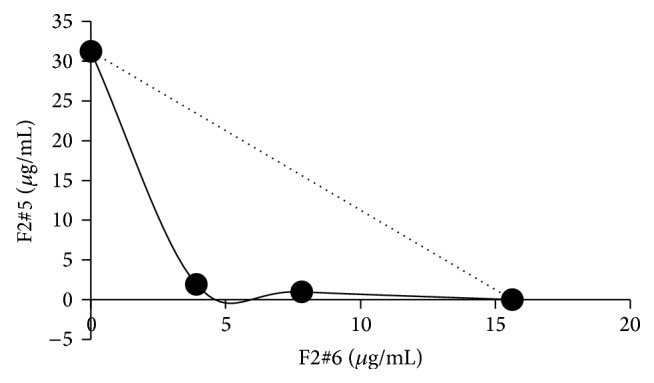
Isobologram showing synergic interaction between F2#5 and F2#6 against* Candida albicans*. FIC = 0.28. The dotted lines indicate a theoretical additive activity. The measured lines yielded a highly concave curve, a characteristic of strong drug synergism (fractional inhibitory concentration index ≤0.5).

In order to verify whether the subfractions could have combinatorial effect among themselves, checkerboard test was performed, and the F2#5 and F2#6 subfractions exhibited synergic activity (FIC 0.28) as shown in Figure 1. Because the subfractions exhibited less activity than F2, the compounds in F2 should act together in a synergistic way for effective antifungal activity. These various substances that act synergistically are known as “phytocomplexes,” in which different substances that do not necessarily have the same mechanism of action, when together, have higher therapeutic efficacy than each isolated substance.

Synergistic effects between CE or each subfraction and fluconazole or nystatin were also evaluated. The CE, F2#5, and F2#6 resulted in a FIC ≤ 0.5 (data not shown), which means, according to Odds [27], that the CE, ethyl acetate fraction, and its subfractions increased the antifungal activity of fluconazole and nystatin (i.e., a synergistic effect).

The CE and its fractions were effective against yeasts that were sensitive or resistant to fluconazole and also to clinical isolates (Table 2). The MICs remained close to those found for the antifungal activity against standard yeast, between 7.8 and 31.3 *μ*g/mL. Fluconazole had a MIC between 3.1 and 12.5 *μ*g/mL against clinical yeast isolates; however, against fluconazole-resistant strains the MIC was >50 *μ*g/mL. These results suggest that derivatives of the plant species* C. americana* exert their effects through mechanisms of action that are different from fluconazole.

*In vitro* cytotoxicity was evaluated in a Vero cell culture and hemolysis assay (Table 3). The cytotoxic concentration (CC_50_) of the CE in Vero cells was 250 *μ*g/mL, showing greater toxicity than their fractions. The F2#5 subfraction had the lowest toxicity against Vero cells (CC_50_ = 714 *μ*g/mL). The evaluation of hemolytic ability in human erythrocytes showed that the extracts and their fractions had low toxicity. Even when considering a concentration of 125 *μ*g/mL for the test drugs, hemolytic activity was observed only with F2 (15.2%), which was lower than amphotericin B (65.8%). When considering the concentration near the MIC (15.6 *μ*g/mL), the highest percentage of hemolysis for F2 was only 5.8%. The presence of tannins and saponins has already been described for* Curatella* species. The tannin was characterized as having a high ability to interact with proteins [35], and saponins can interact with the cell membrane, causing cell disruption [10]. Thus, in-depth evaluations are necessary to ensure the safety of the herbal preparation.

**Figure 2:**
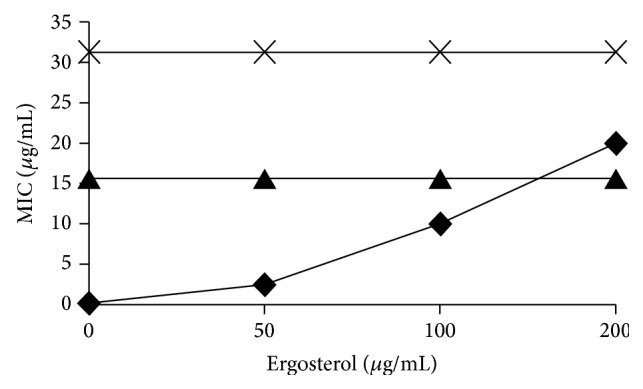
Effect of exogenous ergosterol (0–200 *µ*g/mL) on the MIC of amphotericin B (◆), CE and subfraction F2#6 (▲), and subfraction F2#5 (×) against* C. albicans*.

In evaluating the effect on ergosterol, the CE and F2 subfractions maintained their antifungal effects, even in the presence of different concentrations of ergosterol in the growth medium (Figure 2), suggesting that its mode of action is not the same as amphotericin B. Amphotericin B is a polyene that acts on the induction of membrane permeability to potassium, sodium, hydrogen, and nanoelectrolytes in the phospholipid bilayer and forms a complex with fungal ergosterol located in the membrane, resulting in the leakage of intracellular components and subsequent cell death [36]. Because of substrate competition, the increasing concentration of ergosterol in the medium is inversely proportional to its antifungal activity, meaning that the ergosterol concentration is essential for maintenance of the efficacy of amphotericin B. These results show that the extracts, fractions, and subfractions do not depend on ergosterol for their antifungal action, reinforcing the notion that they have a fungistatic effect.

**Figure 3:**
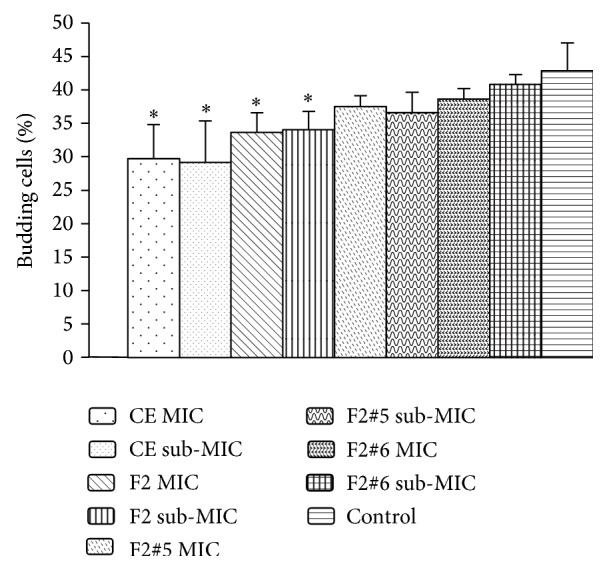
Effect on budding of* C. albicans*. Only the crude extract and the ethyl acetate fraction showed effect on budding. ^*∗*^Statistically significant difference (*p* < 0.05).

To better understand the action of phytocomplexes on yeast, the effects of the CE, fractions, and subfractions on virulence factors of* C. albicans* were evaluated. The virulence of* C. albicans* includes recognition of the host and the binding of yeast to their cells and host cell proteins, and this linkage can be facilitated by the transition between the yeast cell and filamentous growth (morphogenesis) compared with isotropic growth from a single yeast cell. Therefore, the presence of filamentous cells or budding is associated with virulence and pathogenicity [37]. In the test of budding inhibition, we found that the CE and F2 had an inhibitory effect that was significantly different from the control group with regard to the average number of cells with budding (Figure 3). Even at the highest concentration, equivalent to 2x MIC, the CE, fractions, and subfractions showed no effect on germ-tube formation, whereas 6.3 *μ*g/mL nystatin visibly inhibited it.

**Figure 4:**
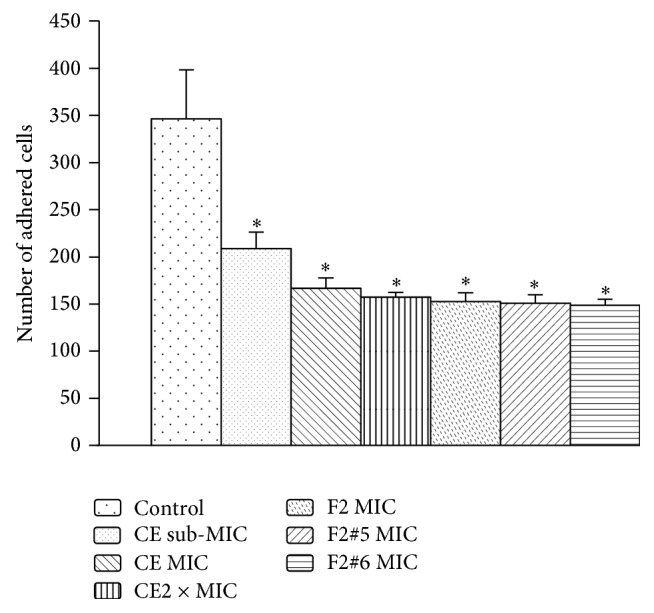
Evaluation of the ability of* C. albicans* to adhere to buccal epithelial cells (BEC). ^*∗*^Statistically significant differences (*p* < 0.05).

**Figure 5:**
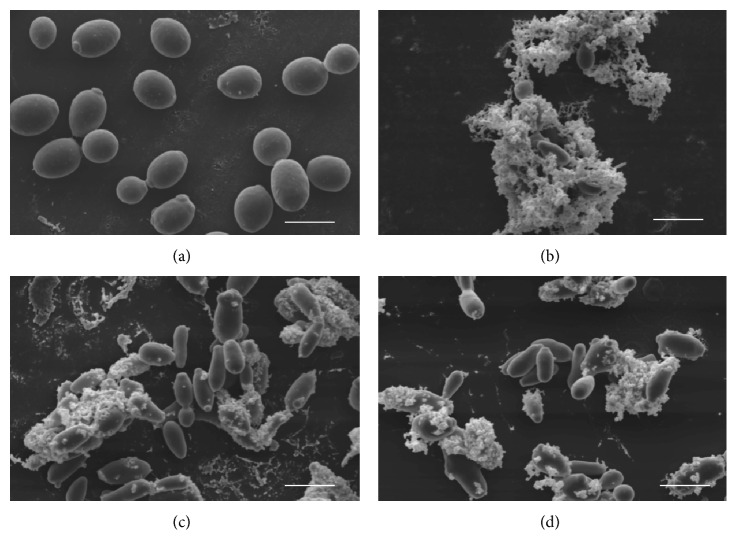
Scanning electron microscopy (Bar: 5 *μ*m; Mag.: 3000x). The morphological alterations observed in treated cells were yeast agglutination and granular matter deposited on the cell walls of the fungi. (a) Control; (b) CE; (c) F2; (d) F2#5 and F2#6 had similar characteristics.

The analysis of the ability of the CE, ethyl acetate fraction, and subfractions at their respective sub-MICs to inhibit adherence on an abiotic surface, with nystatin as a comparison, showed that the number of adhered cells decreased by 98-99%, demonstrating a clear effect on the adherence of yeast to glass slides. All of the samples were able to inhibit the adherence of* C. albicans* in BECs (Figure 4), which was significantly different from the control group. The F2#6 subfraction had the greatest effect, in which the number of yeast cells adhered to BECs decreased approximately 57%, a higher proportion than for the CE at 2x MIC. This demonstrates a reduction of adhesion ability in yeast treated with the drugs derived from plant species* C. americana*, which is an important factor for decreasing the virulence of this yeast. These results are consistent with the theory that tannins bind with proteins in the cell wall of yeast [38, 39]. Scanning electron microscopy suggested that this decrease in adherence may be attributable to the changes observed in treated cells, which form an assemblage between yeast and the deposition of a granular consistency outside the cell walls of fungi (Figure 5). These same ultrastructural characteristics of proanthocyanidins of the plant species* Stryphnodendron adstringens* (Leguminosae) and their tannins can induce modifications of plasmalemma permeability and changes in cytoplasm content, in which the cell wall showed a loss of integrity and low electrodensity [22]. These formations indicate an interaction between the polyphenols present in the CE and its fractions and proteins in the cell wall and could be related to the decreased adhesion ability of* C. albicans*.

Additionally, the hydrophobicity assay revealed that the extract of* C. americana* and its fractions at their respective MICs significantly decreased cell surface hydrophobicity (Table 4). Hydrophobic yeast cells bind profusely in many tissues, whereas a decrease in hydrophobicity reduces the adhesion of yeast [40]. The CE at 4x MIC (62.5 *μ*g/mL), F2 at MIC and 4x MIC (3.2 and 12.8 *μ*g/mL, resp.), F2#5 at MIC and 4x MIC (31.3 and 125.0 *μ*g/mL, resp.), and F2#6 at sub-MIC, MIC, and 4x MIC (7.8, 15.6, and 62.5 *μ*g/mL, resp.) were significantly different from controls (Table 4). The exposure of* C. albicans* to these compounds for 48 h interfered with CSH. The F2#6 subfraction decreased the HI from 95.1% to 84.5% at MIC and 36.2% at 4x MIC (62.5 *μ*g/mL).

**Figure 6:**
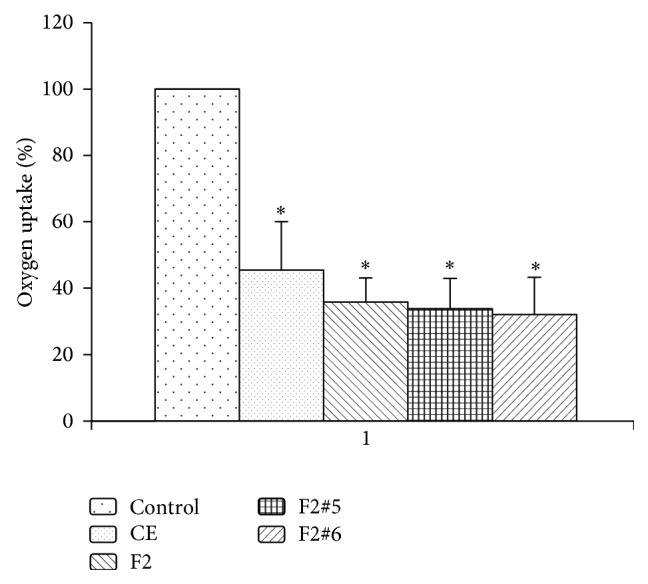
Evaluation of oxygen consumption of* C. albicans* treated with crude extract, fractions, and subfractions. All samples tested showed effective activity, decreasing oxygen consumption by the yeast* C. albicans*. ^*∗*^Statistically significant difference (*p* < 0.05).

The influence on oxygen consumption by yeast was confirmed for extracts and fractions tested (Figure 6). Oxygen is essential for cell division and the maintenance of cell viability. According to Thati et al. [32] and Geraghty and Kavanagh [41] reduced cellular respiration causes a reduction of the synthesis of ergosterol, a steroid that is essential for maintaining membrane integrity and cell division. The F2#5 and F2#6 subfractions caused a greater reduction of oxygen consumption by yeast (33.6% and 31.3% decreases, resp.). The CE and F2 caused 45.4% and 35.2% decreases, respectively. The level of oxygen may limit metabolic regulators, and low oxygen consumption can then reduce the pathogenicity of yeast [42]. This decrease in oxygen consumption may be linked to the mechanism of action [38], in which tannins act directly on the metabolism of the microorganism by inhibiting oxidative phosphorylation.* Herpetomonas samuelpessoai* treated with extracts rich in tannins obtained from* Stryphnodendron adstringens* Mart. presented evident mitochondrial swelling and a reduction of activity of the enzyme succinate cytochrome c reductase, interfering with the energy metabolism of the cell [43]. Transmission electron microscopy indicated that the cell wall of* C. albicans* treated with tannins of the same plant species exhibited a loss of integrity and low electrodensity, inducing modifications of plasmalemma permeability and changes in cytoplasm content, reflected by a difference in the electrodensity of treated cells compared with controls [30].

The present study confirmed the antifungal activity of the ethnomedicinal species* Curatella americana* L. against* Candida* species. Although home preparation has presented a pharmacological effect, it can produce potential toxic effects, indicating that the preparation and use of medicinal plants should be performed under professional supervision. From the active subfractions (F2#5 and F2#6), we were able to isolate the substances 4′-*O*-methyl-catechin (F2#5.4.1), epicatechin-3-*O*-gallate (F2#6.2.1), and 4′-*O*-methyl-catechin-3-*O*-gallate (F2#6.1.2); however, they showed relatively low antifungal activity. This suggests a synergistic action between different substances in the plant drug. If rigorous quality control is performed, then the CE or its fractions could be safe and effective herbal drugs with low production costs. Another important property of the plant species under study is that being able to maintain the activity of the CE and its fractions against fluconazole-sensitive and fluconazole-resistant strains may open new perspectives for alternative treatments.

## Figures and Tables

**Table 1:** Minimum inhibitory concentration (MIC) of crude extract (CE), fractions, subfractions, and isolated compounds from *C. americana*.

Samples	MIC (*µ*g/mL)		
	*C. albicans *	*C. tropicalis *	*C. parapsilosis *
CE	15.6	31.3	31.3
Aqueous fraction (F1)	500.0	500.0	500.0
Ethyl acetate fraction (F2)	3.9	15.6	7.8
F2#5 subfraction	31.3	31.3	15.6
F2#6 subfraction	15.6	31.3	7.8
4′-O-Methyl-catechin (F2#5.4.1)	62.5	125.0	125.0
Epicatechin-3-O-gallate (F2#6.2.1)	62.5	125.0	125.0
4′-O-Methyl-catechin-3-O-gallate	31.3	62.5	62.5
Nystatin	1.6	3.1	1.6

**Table 2:** Minimum inhibitory concentration (MIC) of extracts, fractions, and subfractions against strains of *Candida *spp. fluconazole-sensitive and fluconazole-resistant and from clinical isolates.

Yeasts	MIC (*µ*g/mL)				
	CE	F2	F2#5	F2#6	Fluconazole
*Candida albicans* (LC352)	7.8	7.8	15.6	7.8	12.5
*Candida tropicalis* (LC299)	7.8	7.8	7.8	7.8	6.3
*Candida parapsilosis* (LC144)	7.8	7.8	7.8	7.8	3.1
*Candida albicans* (32res)	15.6	15.6	31.3	15.6	100.0
*Candida albicans* (32Bres)	31.3	31.3	15.6	15.6	>100.0
*Candida albicans* (48res)	15.6	15.6	15.6	15.6	50.0
*Candida albicans* (103res)	15.6	15.6	31.3	15.6	100.0
*Candida albicans* (48sen)	15.6	15.6	31.3	15.6	50.0

**Table 3:** Cytotoxicity and hemolysis assay of crude extract and their fractions.

Samples	CC_50_ (*µ*g/mL)	Hemolytic activity (%)	
		15.6 *μ*g/mL	125 *μ*g/mL
CE	250 ± 12.0	1.4	8.5
F2	650 ± 7.0	5.8	15.2
F2#5	714 ± 20.5	1.1	2.4
F2#6	357 ± 3.5	0.4	2.0
Amphotericin B	—	3.3	65.8

**Table 4:** Effect on cell surface hydrophobicity (CSH) with hydrophobicity index (HI), *Candida albicans *(HI_Control_ = 95.1%  ± 0.8).

Samples	HI (%)		
	Sub-MIC	MIC	4x MIC
CE	94.2 ± 0.7	93.6 ± 0.6	47.2 ± 0.4^*∗*^
F2	96.4 ± 0.8	91.8 ± 0.7^*∗*^	47.4 ± 0.6^*∗*^
F2#5	93.7 ± 1.1	90.1 ± 1.0^*∗*^	39.9 ± 0.6^*∗*^
F2#6	86.7 ± 2.2^*∗*^	84.5 ± 3.1^*∗*^	36.2 ± 1.1^*∗*^

^*∗*^Statistically significant difference (*p* < 0.05).

## Abbreviations

F1: Aqueous fraction
ATCC: American type culture collection
BEC: Buccal epithelial cells
CC_50_: 50% cytotoxic concentration
CE: Crude extract
CFU: Colony-forming units
CPE: Cytopathic effect
CSH: Cell surface hydrophobicity
EC_50_: 50% effective concentration
F2: Ethyl-acetate fraction
F2#: Subfraction of F2
FBS: Fetal bovine serum
FIC: Fractional inhibitory concentration
HSCCC: High-speed countercurrent chromatography
HTO: Tocantins herbarium
MIC: Minimal inhibitory concentration
OD: Optical density
SI: Selectivity index
SSG: Saline solution containing 1% glucose and buffered to pH 7.25
subMIC: Half the MIC
TCID_50_: 50% tissue culture infective dose.
